# Sternal retraction–induced internal mammary artery graft flow impairment: intraoperative assessment and mechanistic insights: a case report

**DOI:** 10.1093/ehjcr/ytag812

**Published:** 2026-11-10

**Authors:** Yukio Umeda, Yuta Inoue, Shohei Mitta, Yukihiro Matsuno, Kenichiro Azuma

**Affiliations:** Department of Cardiovascular and Thoracic Surgery, Gifu Prefectural General Medical Center, Noishiki 4-6-1, Gifu, 500-8717, Japan; Transplantation Research Center, Brigham and Women’s Hospital, Harvard Medical School, 221 Longwood Ave, Boston, MA 02115, USA; Department of Cardiovascular Surgery, Gifu University Graduate School of Medicine, Yanagido 1-1, Gifu, 501-1194, Japan; Department of Cardiovascular and Thoracic Surgery, Gifu Prefectural General Medical Center, Noishiki 4-6-1, Gifu, 500-8717, Japan; Department of Cardiovascular and Thoracic Surgery, Gifu Prefectural General Medical Center, Noishiki 4-6-1, Gifu, 500-8717, Japan; Department of Cardiovascular and Thoracic Surgery, Gifu Prefectural General Medical Center, Noishiki 4-6-1, Gifu, 500-8717, Japan

**Keywords:** Coronary artery bypass grafting, Internal mammary artery, Sternal retraction, Graft flow impairment, Transit-time flow measurement, Thoracic outlet, Case reports

## Abstract

**Background:**

The internal mammary artery (IMA) is the primary conduit for coronary revascularization, making reliable intraoperative flow assessment indispensable. We describe a rare case in which left IMA (LIMA) graft flow was compromised not by conduit or anastomotic issues but by sternal manipulation.

**Case summary:**

A 76-year-old man undergoing emergency coronary artery bypass surgery reproducibly demonstrated markedly reduced LIMA flow only during wide sternal retraction. Intraoperatively, pronounced unilateral sternal retraction was noted, and postoperative computed tomography confirmed first- and second-rib fractures.

**Discussion:**

These findings suggest a dynamic, retraction-dependent inflow disturbance, possibly related to deformation of the thoracic outlet and proximal subclavian artery. This case underscores the need for careful sternal manipulation, which may help avoid unnecessary graft revision or conduit replacement.

Learning points

- Excessive unilateral sternal retraction can cause dynamic, reversible proximal inflow disturbance and reduce left internal mammary artery (LIMA) graft flow, even when the anastomosis is patent.
- Rib injuries associated with internal mammary artery harvesting may contribute to asymmetric chest wall compliance, potentially resulting in thoracic outlet deformation and reduced inflow to the LIMA.
- Unexplained low LIMA graft flow should prompt reassessment after reducing sternal retraction before graft revision is undertaken.
- Persistent LIMA graft flow abnormalities warrant further investigation to identify other potential causes of graft dysfunction.

## Introduction

The internal mammary artery (IMA) is the graft of choice for left anterior descending artery (LAD) revascularization owing to its superior long-term patency and survival benefit.^1^ Accurate intraoperative assessment is therefore essential to confirm graft quality and prevent perioperative ischaemic complications.^2^ Although sternal retraction–associated impairment of IMA graft flow has been reported previously, the anatomical and biomechanical mechanisms responsible for this phenomenon have not been adequately addressed.^3–5^

We describe a case in which LIMA graft flow reproducibly deteriorated during excessive sternal retraction and normalized upon release, providing insights into a possible biomechanical mechanism associated with ‘unilateral sternal retraction’ (see Supplementary material online, *Video S1*).

## Summary figure

**Figure:**
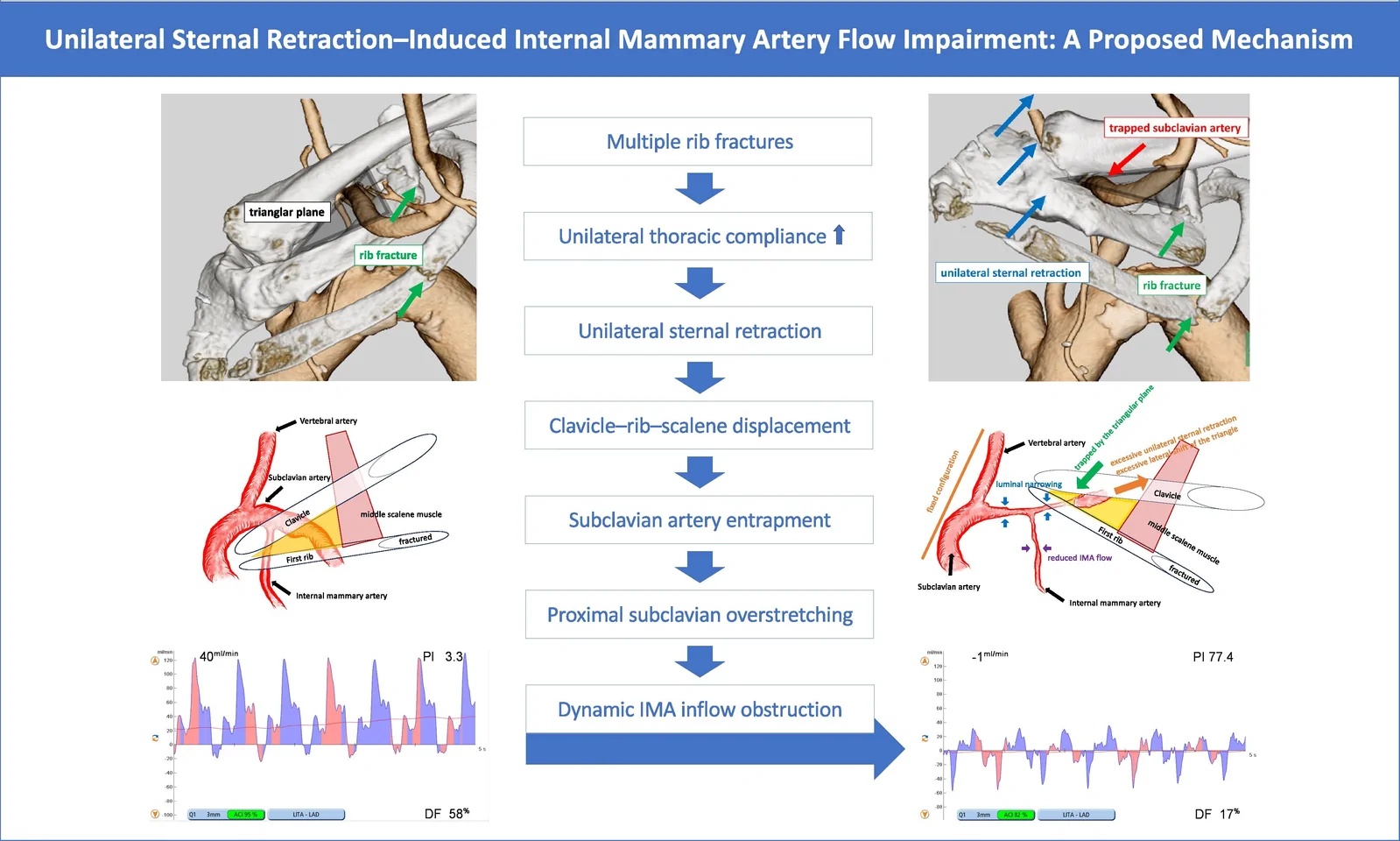


## Case presentation

A 76-year-old man with left main and triple-vessel coronary artery disease underwent emergency coronary artery bypass grafting under cardiopulmonary bypass (CPB). The LIMA was harvested using a takedown-pattern retractor. After an uneventful side-to-side anastomosis to the LAD was completed, transit-time flow measurement (TTFM) demonstrated severely reduced graft flow.

Suspecting a technical problem, we reopened the distal end of the LIMA, but almost no bleeding was observed. We then conducted a systematic evaluation. Antegrade cardioplegia was administered through the aortic root. Brisk efflux of cardioplegic solution from the reopened distal LIMA demonstrated unobstructed passage from the LAD through the side-to-side LIMA-LAD anastomosis, suggesting that the anastomosis was patent and technically satisfactory. Gentle probe passage excluded conduit stenosis. Neither direct stretching of the LIMA pedicle nor conduit kinking was evident on visual inspection. The graft remained appropriately oriented without obvious twisting or positional deformation. In addition, no mediastinal structure capable of causing focal compression of the graft was identified. After these evaluations failed to identify an anastomotic, conduit-related, or mechanical cause of the reduced flow, brisk free flow immediately returned once the sternal retractor was released.

Following reclosure of the distal LIMA, TTFM confirmed adequate graft flow only when the sternum was approximated, whereas excessive retraction consistently produced a characteristic to-and-fro pattern, strongly suggesting a dynamic, retraction-dependent inflow disturbance (*Figure 1*).

**Figure 1:**
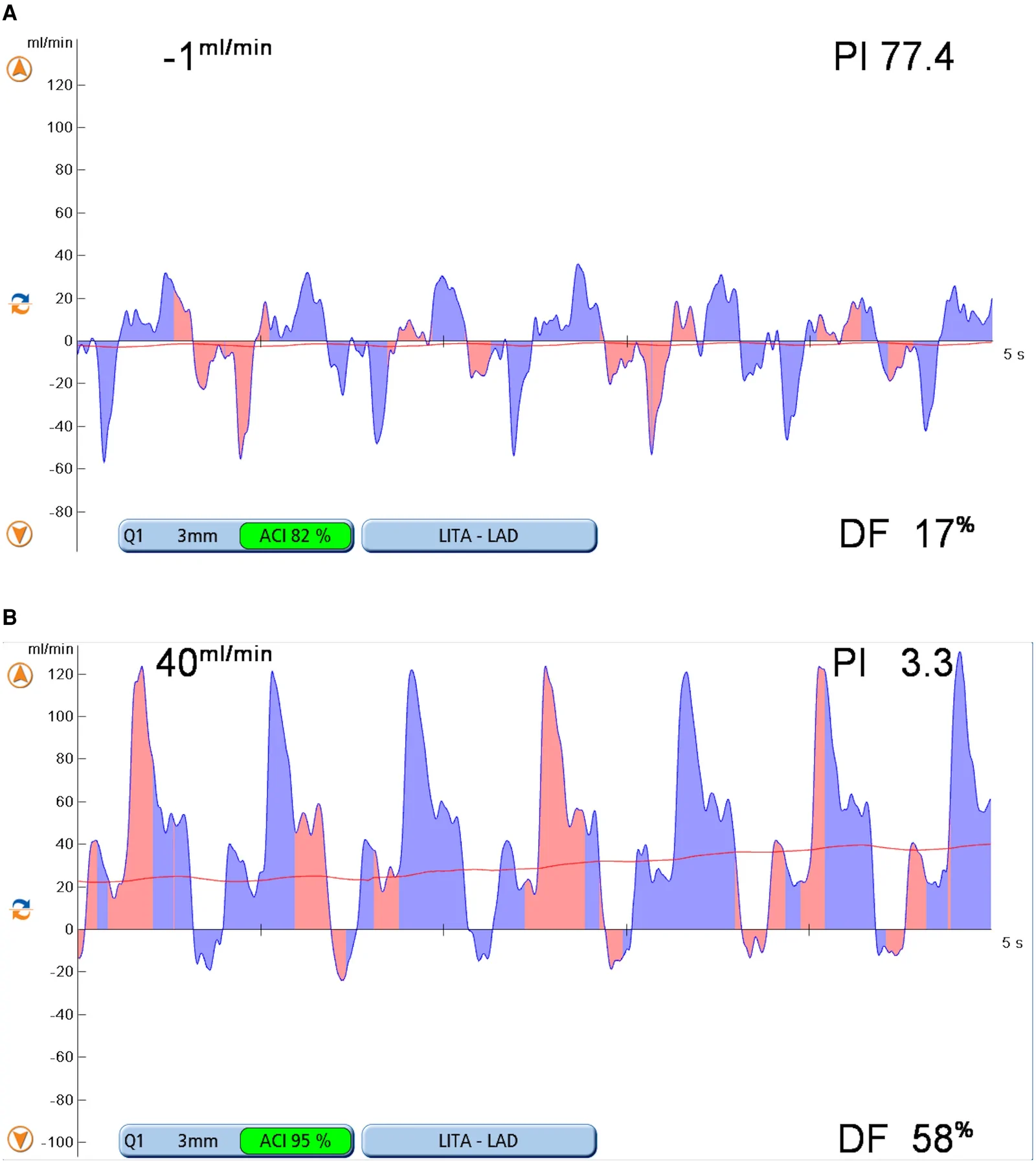
Transit-time flow measurement under excessive sternal retraction (*A*) and moderate sternal retraction (*B*). Under excessive sternal retraction, the LIMA graft demonstrated a characteristic to-and-fro flow pattern with a markedly elevated pulsatility index (PI, 77.4) and severely reduced diastolic filling (DF, 17%) (*A*). In contrast, under moderate sternal retraction, graft flow was 40 mL/min with a PI of 3.3 and DF of 58%, indicating satisfactory graft function (*B*). These findings support a dynamic and retraction-dependent impairment of LIMA inflow. TTFM, transit-time flow measurement; PI, pulsatility index; DF, diastolic filling.

Sequential saphenous vein grafts to the left circumflex artery and posterior descending artery were completed, and the patient was weaned uneventfully from CPB. Postoperative CT angiography confirmed the patency of all grafts.

Intraoperatively, we observed a markedly asymmetric pattern of sternal opening during wide sternal retraction, which we termed ‘unilateral sternal retraction’. Specifically, the right sternal edge exhibited minimal displacement, whereas the left sternal edge showed excessive lateral displacement. As demonstrated in Supplementary material online, *Video S1*, the right sternal edge (blue line) remained nearly stationary despite progressive opening of the retractor, while the left sternal edge (red line) moved markedly laterally. During observation of the left thoracic cavity, a second-rib fracture and buckling of the lower ribs at the costal cartilage were identified. Postoperative computed tomography subsequently revealed an additional fracture of the left first rib. These findings suggested the presence of multiple left-sided rib injuries that may have contributed to asymmetric chest wall compliance and thereby predisposed the sternum to open unevenly during retraction.

## Discussion

Intraoperative LIMA graft flow impairment is a clinically critical situation that requires prompt identification of its underlying cause. Previous reports have shown that wide sternal retraction can impair IMA graft flow intraoperatively.^3–5^ However, these reports primarily described the phenomenon itself, and the anatomical and biomechanical mechanisms responsible for the flow reduction were not elucidated. In the present case, we observed reproducible LIMA flow impairment occurring exclusively during excessive unilateral sternal retraction despite an intact conduit and a technically satisfactory anastomosis.

Recognition of multiple left-sided rib injuries provided a plausible anatomical explanation for the observed phenomenon. Rib fractures after internal mammary artery harvesting are well documented and occur frequently.^6,7^ Consistent with previous reports describing an incidence of ∼30%, postoperative computed tomography at our institution demonstrated rib fractures on the IMA-harvest side in 34% of patients undergoing LIMA harvesting. Takedown-pattern retractors, which apply screw-driven rib-elevating forces, simultaneously exert substantial bending stress on the ribs and are therefore particularly prone to inducing such injuries (see Supplementary material online, *Video S2*). Therefore, the rib injuries observed in the present case were considered most likely related to LIMA harvesting and the associated retractor forces.

We hypothesize that the combination of the intraoperatively identified second-rib fracture and buckling of the lower costal cartilages, together with the first-rib fracture subsequently demonstrated on postoperative CT, increased compliance of the left chest wall. This asymmetric increase in compliance may have predisposed the sternum to open unevenly during retraction, resulting in the phenomenon we termed ‘unilateral sternal retraction’, characterized by minimal displacement of the right sternal edge and excessive displacement of the left sternal edge. As demonstrated in Supplementary material online, *Video S1*, progressive opening of the retractor resulted in little movement of the right sternal edge, whereas the left sternal edge exhibited marked lateral displacement.

This abnormal opening pattern has previously been implicated in neurological complications such as brachial plexus injury and Horner syndrome.^6,7^ To our knowledge, however, it has not previously been linked to LIMA flow impairment. This distinction is clinically relevant because earlier reports focused on neurological sequelae, whereas the present mechanism may compromise the haemodynamic integrity of a coronary bypass graft.

We further speculate that unilateral sternal retraction altered the geometry of the left thoracic outlet, consisting of the clavicle, first rib, and middle scalene muscle through which the subclavian artery passes. Excessive displacement of this anatomical complex may have resulted in stretching, torsion, or transient compression of the subclavian artery. Because the proximal subclavian artery is relatively fixed by its origin from the aortic arch and major branch vessels, while the distal portion traverses the thoracic outlet, exaggerated thoracic outlet deformation could potentially generate a functional two-point fixation system, leading to dynamic luminal narrowing and reduced inflow to the LIMA.

In the present case, transit-time flow measurement demonstrated immediate normalization of graft flow following release of the excessive retraction. Likewise, free flow from the distal LIMA promptly recovered once the retractor was released. These observations support the presence of a reversible and retraction-dependent inflow disturbance rather than a fixed conduit or anastomotic abnormality.

Alternative mechanisms should also be considered. Wide sternal retraction may directly stretch the LIMA pedicle, induce transient graft kinking, alter mediastinal geometry, or deform the conduit itself.^3–5^ Because no direct dynamic vascular imaging was performed, the relative contribution of these mechanisms cannot be determined. Nevertheless, the pronounced unilateral nature of the sternal opening, the presence of multiple left-sided rib injuries, and the immediate restoration of flow after release of the retractor suggest that asymmetric thoracic deformation may have played an important role in this case.

Several limitations should be acknowledged. First, this report describes a single case. Second, no dynamic vascular imaging or direct assessment of the subclavian artery was performed during sternal retraction. Therefore, subclavian compression, torsion, or luminal narrowing could not be directly confirmed. Third, the proposed relationship among rib fractures, asymmetric chest wall compliance, unilateral sternal retraction, thoracic outlet deformation, and LIMA inflow impairment remains inferential. Accordingly, the mechanism described herein should be regarded as a plausible biomechanical hypothesis rather than a definitively proven causal pathway.

Taken together, this case highlights a potential link between rib injury associated with LIMA harvesting, asymmetric chest wall mechanics, unilateral sternal retraction, thoracic outlet deformation, and reversible LIMA inflow impairment. Recognition of this possibility may help surgeons avoid unnecessary graft revision, conduit replacement, or inappropriate troubleshooting when confronted with unexplained intraoperative deterioration of LIMA graft flow during wide sternal retraction.

## Summary of the possible mechanism of LIMA flow impairment in this case

- Multiple rib fractures induced by the takedown-pattern retractor
- Increased thoracic compliance on the injured (left) side
- Unilateral sternal retraction during chest opening
- Excessive displacement of the clavicle–first rib–middle scalene muscle triangle
- Possible entrapment of the left subclavian artery within the thoracic outlet triangle
- Possible overstretching and/or torsion of the proximal subclavian artery
- Dynamic inflow obstruction of the LIMA, despite a normal conduit and anastomosis

## Lead author biography


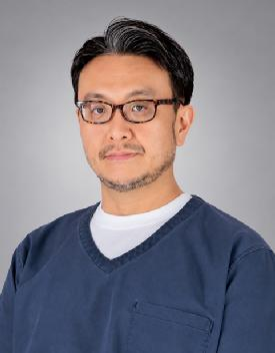


Yukio Umeda, MD, PhD, is Chief of Cardiovascular Surgery at Gifu Prefectural General Medical Center, Japan. His clinical specialties include adult cardiac, aortic, and peripheral vascular surgery. He has developed and introduced novel surgical techniques, including the Tailored Stand-up Collar (TSC) technique for acute aortic dissection and the Rafting Anchor Patch (RAP) repair for left ventricular free-wall rupture. Dr Umeda holds board certifications in surgery, cardiovascular surgery, cardiology, angiology, and general thoracic surgery. He also serves as a Councillor of the Japan Association for Thoracic Surgery.

## Supplementary Material

ytag812_Supplementary_Data

## Data Availability

The data underlying this article cannot be shared publicly because they contain information that could compromise patient privacy. Additional anonymized data will be shared on reasonable request to the corresponding author.

## Notes

**Consent:** The authors confirm that written consent for submission and publication of this case report, including images and associated text, has been obtained from the patient in accordance with COPE guidance.
